# Nondestructive Classification of Maize Moldy Seeds by Hyperspectral Imaging and Optimal Machine Learning Algorithms

**DOI:** 10.3390/s22166064

**Published:** 2022-08-13

**Authors:** Yating Hu, Zhi Wang, Xiaofeng Li, Lei Li, Xigang Wang, Yanlin Wei

**Affiliations:** 1 College of Information Technology, Jilin Agricultural University, Changchun 130118, China; 2 Northeast Institute of Geography and Agroecology, Chinese Academy of Sciences, Changchun 130102, China; 3 Changchun Jingyuetan Remote Sensing Test Site, Chinese Academy of Sciences, Changchun 130102, China; 4 College of Geoexploration Science and Technology, Jilin University, Changchun 130026, China

**Keywords:** hyperspectral imaging, sparrow search algorithm (SSA), random forest (RF), maize mildew, nondestructive detection

## Abstract

Mildew of maize seeds may affect their germination rates and reduce crop quality. It is crucial to classify maize seeds efficiently and without destroying their original structure. This study aimed to establish hyperspectral datasets using hyperspectral imaging (HSI) of maize seeds with different degrees of mildew and then classify them using spectral characteristics and machine learning algorithms. Initially, the images were processed with Otus and morphological operations. Each seed’s spectral features were extracted based on its coding, its edge, region of interest (ROI), and original pixel coding. Random forest (RF) models were optimized using the sparrow search algorithm (SSA), which is incapable of escaping the local optimum; hence, it was optimized using a modified reverse sparrow search algorithm (JYSSA) strategy. This reverse strategy selects the top 10% as the elite group, allowing us to escape from local optima while simultaneously expanding the range of the sparrow search algorithm’s optimal solution. Finally, the JYSSA-RF algorithm was applied to the validation set, with 96% classification accuracy, 100% precision, and a 93% recall rate. This study provides novel ideas for future nondestructive detection of seeds and moldy seed selection by combining hyperspectral imaging and JYSSA algorithms based on optimized RF.

## 1. Introduction

Corn has a reputation as a “golden crop”; even though the seed is small, the crop is vital to China and plays a major role in the international trade in corn seeds. It is of great significance that there are independent and controllable seed sources in the seed industry. High temperature and humidity cause mold to grow on seeds, reducing their germination rates, as well as their quality and nutritional value [1,2]. Over the past few decades, crop diseases have been a frequent cause of crop yield reduction, and their cause must be determined by studying the diseased seeds [3,4]. The seed industry must speed up the promotion of corn seed science, perform efficient seed discrimination, achieve independent self-improvement, and be able to control seed quality independently. Chemical composition analysis is the approach that provides the most precise indication of the level of mold present [5]; however, there is inevitably some damage to the sample, as well as subjective considerations, in the process of analysis [6]. A new and innovative technological tool has emerged in recent years for the nondestructive testing of seeds, known as hyperspectral imaging [7,8,9,10]. Hyperspectral remote sensing imaging (HRSI) uses spectral signatures to identify, detect, and discriminate between objects of varying spectral characteristics [11]. The results are directly proportional to the spectral resolution of the sensor and how much information is stored in each band [12]. Sensors with high resolution tend to have bands that are much tighter than those with low resolution. HRSI is based on a narrow band that combines spatial information and hundreds of channels of spectral information so that the chemical and structural information of seeds may be combined using this technology, which can be used for both extracting aberrant information and determining its spatial distribution [13,14]. A machine learning algorithm can be constructed to categorize crops by using multispectral and multi-temporal images. Both anomalous information and spatial distribution may be obtained if one takes the initiative. On the one hand, the sample does not have to be destroyed during the experiment, so the method is both efficient and nondestructive. On the other hand, the image information from the imaging spectrometer offers research assistance for computer vision. The first consideration is the choice of the featured wavelength. Yang Sai et al. [15], to identify corn seeds, employed a joint skewness technique to select feature wavelengths, and when this was paired with a support vector machine, the model’s classification accuracy increased to 96.28%. However, the value of the skewness distribution is more affected by symmetrical distributions on both sides of the distribution. A one-way tailspin is a tail that spins in one direction. Positive (negative) skewness indicates the tail’s direction of rotation more than its tendency to spin [16]. The second consideration is the selection of the classification algorithm. The hyperspectral imaging distinction and linear discriminant analysis performed by Ali Mohammadi F et al. [17] correctly classified three different types of maize kernels with an accuracy of 95%. After using the watershed technique to partially segment moldy peanuts, Jiang et al. [18] determined the classification impact from their data. Yuan et al. [19] used a support vector machine (SVM), partial least squares discriminant analysis (PLS-DA), and a cluster-independent pattern classifier (SIMCA). Both used dimensionality reduction data and then directly applied machine learning models for classification.

Nevertheless, an RF classification model becomes an optimization problem when the wavelengths of hyperspectral light are divided by a large number. The grid search is straightforward to use, and all combinations of discrete parameter spaces can be evaluated as quickly as possible. It is necessary to discretize continuous parameters before using them [20]. However, the general simulated annealing algorithm is used by other researchers for its ability to search iteratively for optimal parameters. Compared to the initial value, generally simulated annealing (GSA) has a slower convergence speed [21]. The advantage of the swarm intelligence algorithm in the optimization model is highlighted due to the outstanding flexibility of sparrow (sparrow, S) established by Xue et al. [22], who developed a novel swarm intelligence optimization algorithm based on its discovery and contention strategy. In order to simplify the search procedure and avoid anomalies caused by discrete data, the SSA was adopted. The normal distribution was directly used in the search algorithm to ensure continuity. Taking advantage of the SSA simplifies search procedures and eliminates anomalies caused by discrete data. The search algorithm is directly based on normal distributions to maintain continuity. However, the SSA algorithm is prone to judging the local optimum as the optimal global solution [23]. The SSA algorithm continues to be discussed and improved by researchers to improve its performance. As an extension of the basis and model of the SSA, Tang et al. implemented a fusion of the SSA and bird swarm algorithm [24]. The introduction of updated algorithms resulted in the need to update too many position formulae. This paper uses the random forest (RF) model for hyperspectral wavelength importance analysis to extract feature wavelengths. These wavelengths were input into the RF model to create classification models for maize seeds with different degrees of mold. Last but not least, the SSA was used to optimize a machine learning classifier to process hyperspectral data from the perspective of a model. The elite inverse-strategy-enhanced sparrow search algorithm (JYSSA) was used to broaden the search range, maximize the number of forests and feature subsets in the random forest classifier, and search for each of their optimal solutions. This is the first time that a novel approach has been used in the area of nondestructive testing to address the issue of choosing RF model parameters from a large variety of wavelengths, an issue that plays a significant role in the exploration of efficient and comprehensive methods of detecting mold in maize seeds of various ages.

## 2. Materials and Methods

### 2.1. Hyperspectral Imaging and Data Acquisition

**Figure 1:**
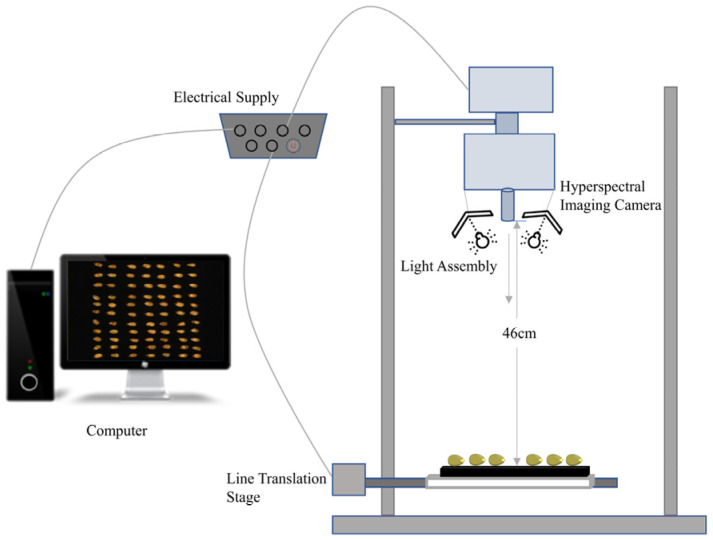
Hyperspectral imaging system.

The system consisted of a Resonon hyperspectral imager (Pika XC2, Resonon Inc., Bozeman, MT, USA) and a computer equipped with data acquisition and control using display software (SpectrononPro, Resonon Inc., USA). The imaging spectrometer has a spectral range of 400–1000 nm, a 50 μm slit, a spectral resolution of 1.3 nm, and a spatial resolution of 0.15 mm/pixel. The other three important components of the entire hyperspectral system are the mount, light source, and camera, as shown in Figure 1. One of the keys to the linear scanning of the hyperspectral imaging instrument is the linear mover, which moves at a uniform rate of 500 steps.

The second essential component is the light source. A sufficient and smooth light source plays a crucial role in a hyperspectral imaging system. A combination of an illumination unit (OSRAM, Munich, Germany) and a 4-lamp illumination system (35 W per lamp, for a total input power of 140 W and a total radiated power of about 5–7 W) provided the light source for hyperspectral imaging (XENOPLAN, F/1.4 FL23 mm, Schneider-Kroetsch, Bad Kroetsch, Germany), and the health sample was placed on a matte cloth. The optimal parameters of the swept spectra were adjusted on a plate-on-mount table with an extinction cloth as follows: object distance of 13.5 cm, exposure time of 7 ms, linear translation table moving at 3.4 mmsl, wavelength range of 400–1000 nm, spectral resolution of 1.3 nm, and 462 bands of scanning hyperspectral images.

The Jilin Academy of Agricultural Sciences supplied the maize seeds. As described in [25], we divided the corn seeds into five groups based on the amount of mold coverage (Table 1) and then placed them on five square plates covered with matting cloth [26]. For smooth irradiation of maize seeds, the instrument needed to be preheated before scanning the seeds.

### 2.2. Image Processing and Spectral Extraction

**Figure 2:**
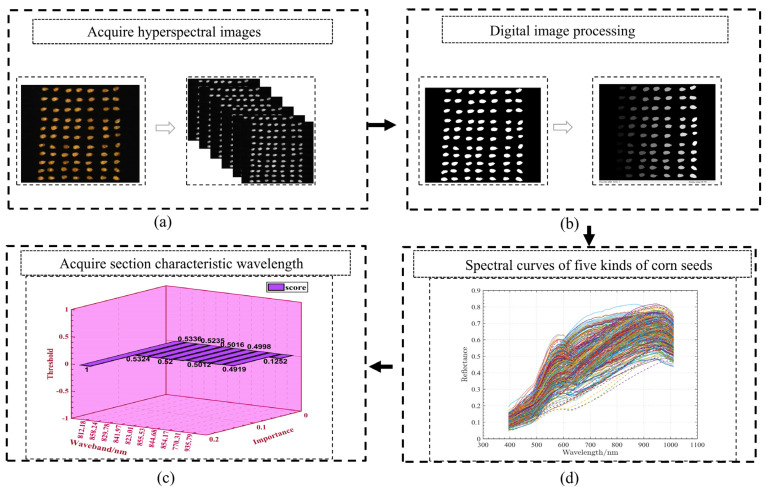
Hyperspectral image processing and curves.

Digital image processing techniques were used to extract the spectral data of the edges of each corn seed, as shown in Figure 2. This was caused by reflection from the sample surface. For the optical darkroom (all of the labs were treated with matte cloth), (a) the blackboard- and whiteboard-corrected images were acquired using a masking lens and standard whiteboard acquisition before scanning (598.71 nm wavelength was the clearest), and (b) applying the Otus threshold method to the 598.71 nm image revealed a clear sample. Next, morphological opening and closing operations were used to establish the sample edges, and the samples were numbered according to the pixels along the edges. (c) By automating the selection of separate hyperspectral bands using masks determined by the numbering and annotation of ROIs, the identification of multiple bands could be achieved. (d) Finally, the wavelength features were implemented across all samples. After the ROI process had been conducted, the radiation values of each pixel point were corrected and recalibrated by referring to the calibration calculation formula, which is defined as follows:

(1) $$C_{S}=\frac{C_{R}-C_{D}}{C_{w}-C_{D}}$$

where $C_{S}$ indicates the calibrated image information, $C_{w}$ is the whiteboard information, and $C_{D}$ is the all-black image information when the dark current is acquired. We found that all of the pixels in each seed were averaged and transformed into the hyperspectral reflectance data of each corn seed, and the corresponding spectral data were extracted in the order of the corn seed numbers.

**Figure 3:**
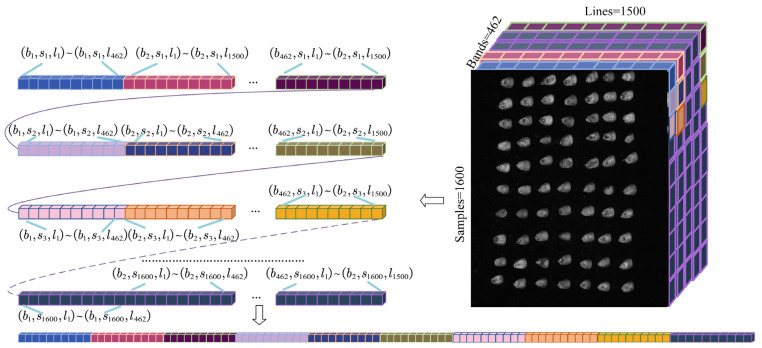
Hyperspectral image processing and curves.

For better presentation of the three-dimensional data, these data are converted into two-dimensional data to make it more obvious. As depicted in Figure 3, the scanning of maize seeds through a hyperspectral imaging system generates three-dimensional images consisting of 1500 lines, 1600 samples, and 462 bands. Each band’s arrangement can be better understood by using binary expansion.

### 2.3. JYSSA Algorithm to Optimize the RF Mode

**Figure 4:**
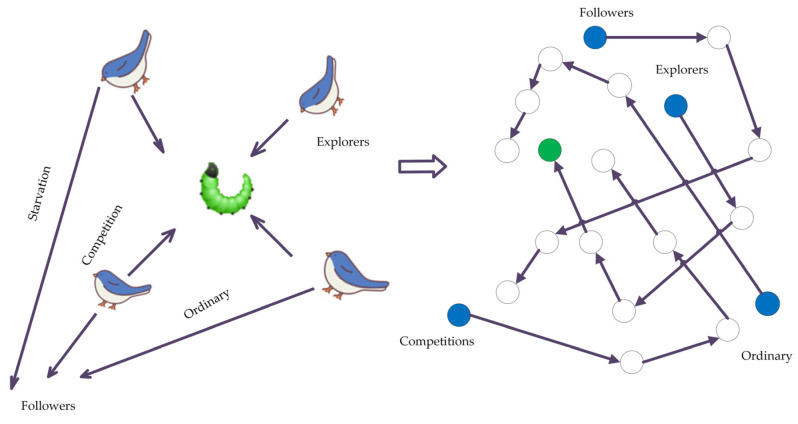
Sparrow search algorithm procedures.

A traditional SSA divides S into three categories based on their energy levels during foraging: explorers, followers (starvation, competition, and ordinary gradual followers), and probers. In the subsection on the S search process, it can be seen that the finder is in the middle and the first to find food from a safe position, as shown in Figure 4.

The SSA algorithm is relatively robust, with simple role assignments and few parameters. However, precisely because of the small number of roles and the lack of chain mechanism in the process of cyclic update of positions, it is prone to local optimum, which is considered to give search results instead of the global optimum, and the global optimum is replaced by the local optimum [27,28].

In terms of initialized populations, the SSA algorithm is improved by adding the elite reverse strategy, the selection of 10% of S as elite S, and the forward and reverse solutions that enable the algorithm to reach more exploration points, effectively eliminating the unknown nature of the algorithm due to random assignment of initial populations, suppressing the algorithm from falling into local optima, and improving its convergence speed.

The search range increases, as usual, which means an ordinary S to choose into an elite S should raise their energy level and expand their foraging abilities, making an explorer S and followers ordinary S compete internally for elite S qualification, the typical follower S does not constantly look for food sources in comparison to their own higher energy explorer S, but instead jumps out of the explorer’s foraging judgment and falls into the local extreme value point, to improve the overall algorithm of global searchability, the degree of accuracy has been increased.

The explorer mainly finds food from the forward and reverse search when updating the position according to Equation (2).

(2) $$X_{i,j}^{t+1}=\{\begin{array}{ll} X_{i,j}^{t}\cdot \exp\left(\frac{-i}{\alpha \cdot iter_{\max}}\right) & ,if\;R_{2}<ST\\ X_{i,j}^{t}+Q\cdot L & ,if\;R_{2}\geq ST \end{array}$$

where *t* denotes the iteration counter, and *Q* is a random number satisfying a normal distribution.

The introduction of the reverse solution allows the explorer to conduct a large-scale search: when $R_{2}\leq ST$, a predator approaches some S, an alarm signal is immediately issued, and all S quickly fly to other safe areas.

Followers, due to their low energy cannot perform foraging movements and can only be constantly supervised by the explorer. When the explorer finds food, signals will be fed back to the followers, and they will immediately update their position according to Equation (3) to plunder food, which is an opportunity to become an explorer.

(3) $$X_{i,j}^{t+1}=\{\begin{array}{cc} Q\cdot \exp\left(\frac{X_{worst}^{t}-X_{i,j}^{t}}{i^{2}}\right) & ,if\;i>n/2\\ X_{p}^{t+1}+|X_{i,j}^{t}-X_{p}^{t+1}|\cdot A^{+}\cdot L & ,otherwise \end{array}$$

where $X_{p}$ is the population center safety position. $X_{worst}^{}$ denotes the current global worst position. *A* denotes the 1 × *d* matrix in which each element is randomly assigned by 1 or −1, the; when i>n/2, it indicates that the first *i* follower with poor health value is most likely to be in the starvation state.

Among them, spotters account for 10–20% of the population size, and their initial positions are randomized. When the natural enemy raided, the spotters immediately updated their positions and gave warning signals according to the following equation

(4) $$X_{i,j}^{t+1}=\{\begin{array}{cc} X_{best}^{t}+\beta \cdot |X_{i,j}^{t}-X_{best}^{t}| & ,if\;f_{i}>f_{g}\\ X_{i,j}^{t}+K\cdot \left(\frac{\left|X_{i,j}^{t}-X_{worst}^{t}\right|}{(f_{i}-f_{w})+\varepsilon }\right) & ,if\;f_{i}=f_{g} \end{array}$$

where $X_{best}^{}$ is the current global optimal position. β is the step control parameter from a normal distribution of random numbers with a mean and variance of 1. *K* is the moving step of a random number. $f_{w}$ is the worst adaptation value. $f_{g}$ is the optimal fitness value. *ε* is the smallest constant that circumvents the absolute zero point.

**Figure 5:**
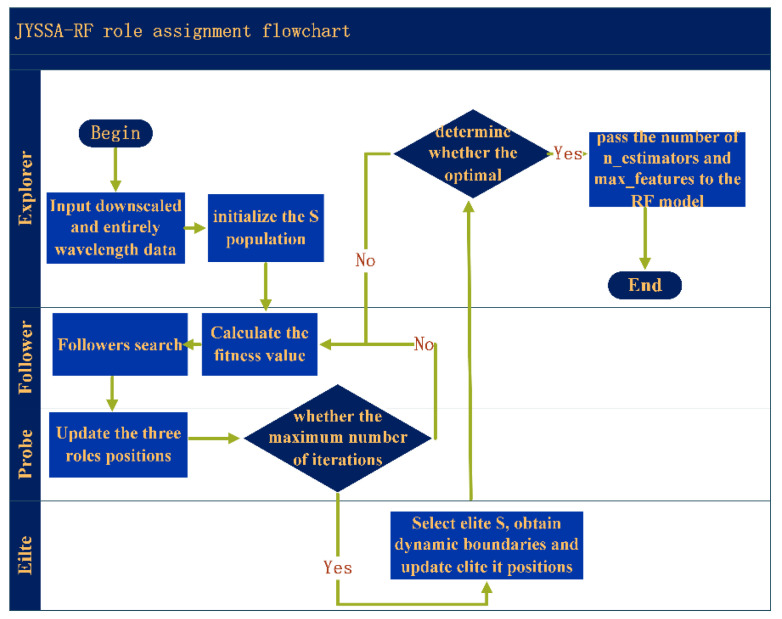
JYSSA algorithm procedures.

The JYSSA algorithm optimizes the RF classification model, as shown in Figure 5, with the following flow.

(1) Full wavelength with feature selected band as input with input sizes of 462 and 186.
(2) Initialize the S population to assign explorers and followers and iterate through the loop by searching the S population’s search range in reverse.
(3) Calculate the fitness value for each individual, ranked in order of high and low.
(4) Update the explorer, follower, and probe positions according to Equations (2)–(4).
(5) Calculate the fitness value again and reorder it, determine whether the maximum number of iterations and the expected convergence effect are satisfied, and if so, continue to the next step; otherwise return to (3).
(6) Select elite S, obtain dynamic boundaries, and update elite S positions using the elite reversal strategy.
(7) Update the fitness value again, determine whether the optimal individual is found, and pass the number of trees and feature subsets to the RF model if found; otherwise, repeat Steps (2)–(6).

### 2.4. Model Prediction and Testing 

The training and test sets were divided hyperspectrally into $C=\left\{\left(corn_{1},label_{1}),\ldots ,(corn_{n},label_{n}\right)\right\}$, where the test set data were preprocessed with *n* data, representing the total number of bands. In the SSA-optimized RF model, $corn_{n}\;\mathrm{and}\;label_{n}$ represent the spectral features of maize seeds and the authenticity labels for those seeds, respectively.

For this paper, accuracy, precision, and recall were used to evaluate the results of the classification. The testing sets were classified by the research method only if they gave the same classification result as the pre-classified results; otherwise, the classification was considered incorrect. The formulae of precision, recall, and evaluation index are as follows:

(5) $$\begin{array}{c} R_{n}=\frac{C_{n}}{T_{n}}\\ Accuracy=\frac{C_{p}}{C_{m}} \end{array}$$

where $C_{n}$ indicates that the label is in class *n*, and classified as *n* seeds of corn labeled as a class; *S_n_* denotes the total number of corn seeds labeled as *n*; *T_n_* denotes the true label; *n* denotes the total number of corn seeds in the test set with the true label; $C_{p}$ denotes the total number of correctly predicted classes for the entire process *p* of the total number of maize seeds; and $C_{m}$ denotes the total number of maize seeds in the entire dataset.

## 3. Results and Discussion

### 3.1. Analysis of Spectral Curves of Maize Seeds with Different Degrees of Mildew

**Figure 6:**
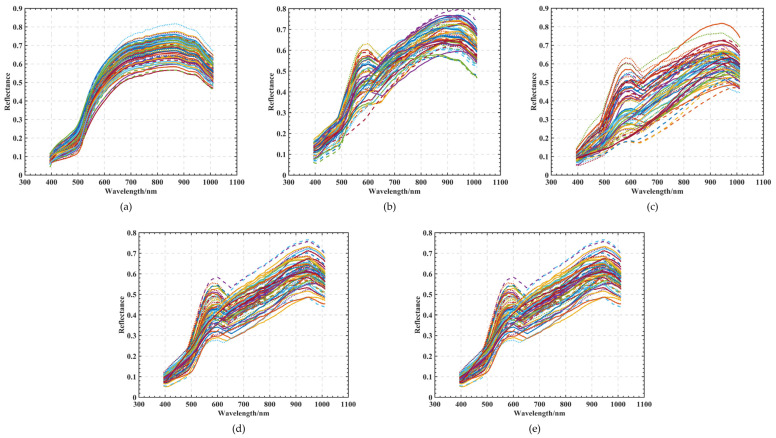
Spectral curves of five kinds of maize seeds with different degrees of mildew: (**a**) A1 maize seed spectral curve; (**b**) A2 maize seed spectral curve; (**c**) A3 maize seed spectral curve; (**d**) A4 maize seed spectral curve; (**e**) A5 maize seed spectral curve.

Using the hyperspectral imaging system, curve images were obtained with different degrees of hyperspectral characteristics for five classes of maize seeds, totaling 60 grains for each class. The spectral curves of each maize seed in the five classes of seeds were corrected according to the digital image processing described in Section 2.1 Figure 6a shows the spectral curve of healthy seeds, which is used as a criterion for judging moldy seeds. Figure 6b shows the phenomenon of wave peaks between 500 and 700 nm, and the curve is different from that shown in Figure 6a. In Figure 6c–e, the reflectance gradually decreases from 500 to 700 and 700 to 900 nm; mold between the corn seeds causes the absorption of light, and the reflectance of the wave crest gradually decreases.

In addition to SNV, MSC, smoothing, etc., the first derivative and second derivative were used to process the spectral curves. In order to calibrate the standard normal variate (SNV), each wavelength point’s absorbance value must fall into a certain distribution along the spectral curve. The rows of the spectral matrix determine which spectrum to handle using the SVN. In short, multiplicative scatter correction (MSC) eliminates scattering losses caused by uneven particle size distributions and uneven particle distributions. In order to minimize spectral differences, MSC tries to preserve as much chemical-related information as possible throughout the spectrum. Based on the algorithm, the wavelength and sample concentration are not taken into account when calculating the scattering. Instead of preprocessing a single curve, MSC and SVN preprocess sets of sample curves.

**Figure 7:**
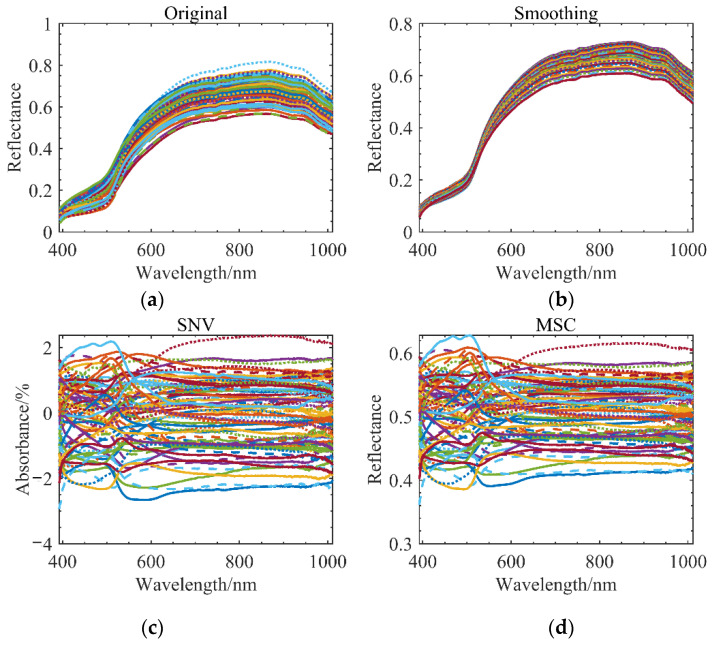
Spectral curve preprocessing: (**a**) original spectral curve; (**b**) spectral curve after smoothing; (**c**) spectral curve after SVN; (**d**) spectral curve after MSC.

Since the first and second derivatives are obtained after taking the derivatives of the curves, background interference is eliminated, and the resolution is improved. The derivative can increase the resolution and the number of wavelength sampling points, but it amplifies the noise and reduces the signal-to-noise ratio when processing high-frequency noise. From Figure 7, it can be seen that the MSC and SVN algorithms were both used concurrently to preprocess all kinds of maize mildew degree curves. Instead of preprocessing a single curve, MSC preprocesses a set of sample curves; a single spectral curve is best preprocessed with SVN. 

### 3.2. Data Dimensionality Reduction and Feature Selection

**Figure 8:**
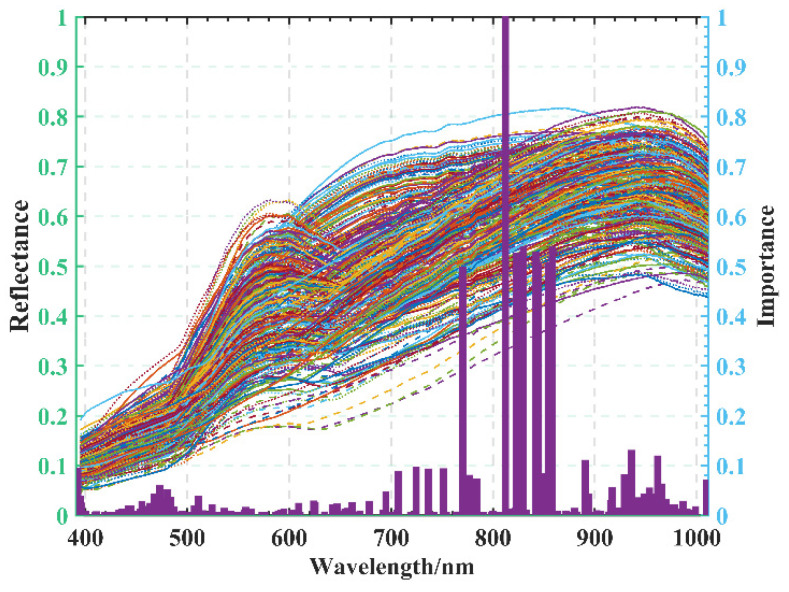
Spectral features are important.

The hyperspectral imaging system provides a wealth of data, but when the number of band operations increases, the training time also increases because the band attribute values are too low, reducing the accuracy [29]. For this reason, RF features and importance ranking were used. All bands were given their scores, facilitating the screening of high-priority bands. Figure 8 mainly shows the 186 feature bands with importance scores greater than 0, which were screened in the experiment for comparison with the full 462 bands.

**Figure 9:**
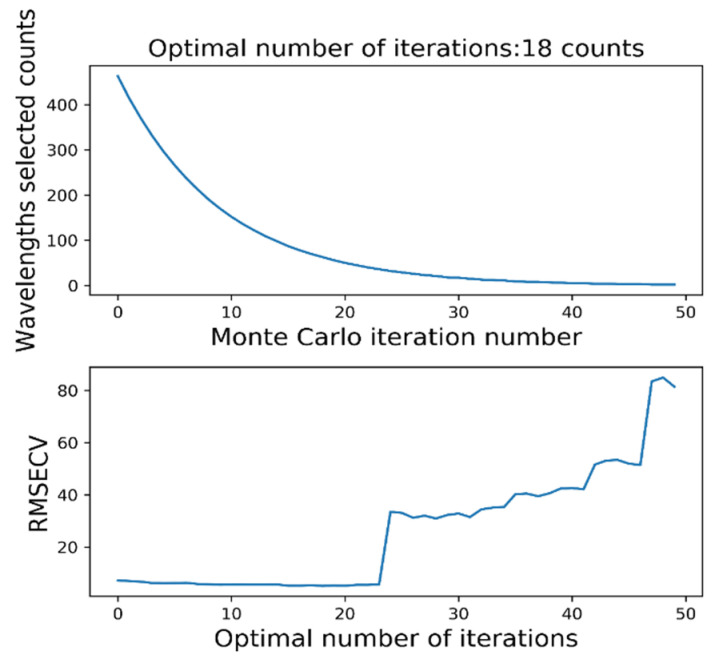
CARS algorithm’s feature band selection.

Feature bands were selected based on preprocessed data. As part of the selection process, competitive adaptive reweighted sampling (CARS) was adopted, which relies primarily on Monte Carlo analysis and PLS regression to find the feature wavelengths. After continuous PLS cross-validation (RMSECV) shrinking, a maximum root-mean-square error characteristic wavelength subset was found, and the PLS model was re-established through the new subset according to Darwin’s theory of evolution. There were eight training sets and two test sets in the wavelength dataset, and the number of Monte Carlo samplings was fixed. In Figure 9, the absolute weight of the regression coefficient is shown for each sampling process. When determining the optimal characteristic wavelengths, small absolute wavelengths were discarded directly through the decreasing function (EDF) and then cross-validated to minimize the RMSECV.

### 3.3. Optimal Model Parameters

**Figure 10:**
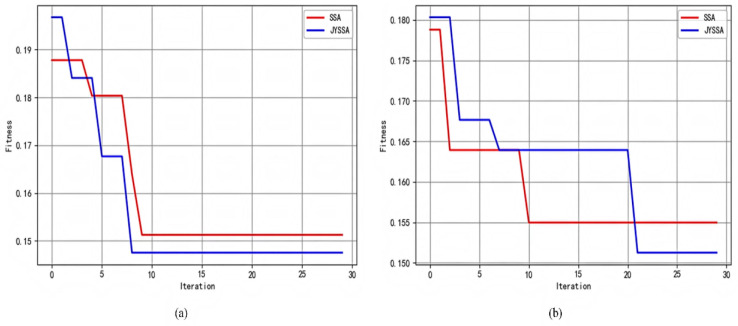
Convergence of algorithm adaptation curves: (**a**) convergence curves of 462 bands; (**b**) convergence curves of 186 bands.

There is a clear trend that the SSA jumps out of the local optimum but does not continue to search for the optimal solution, while the JYSSA algorithm continues to decline after jumping out of the local optimum, and its ability to jump out of the local optimum and search for the global optimum is improved compared with the original algorithm. Table 2 and Table 3 optimize parameters between different algorithms for band 462 and band 186, respectively. Figure 10 compares the convergence and degree of adaptation of the SSA and JYSSA algorithms. The optimal adaptation degree is taken to evaluate the performance of the algorithm, and the lower the optimal adaptation degree, the better the algorithm’s effect [30]. 

### 3.4. Comparison of Classification Models and Experimental Results 

**Figure 11:**
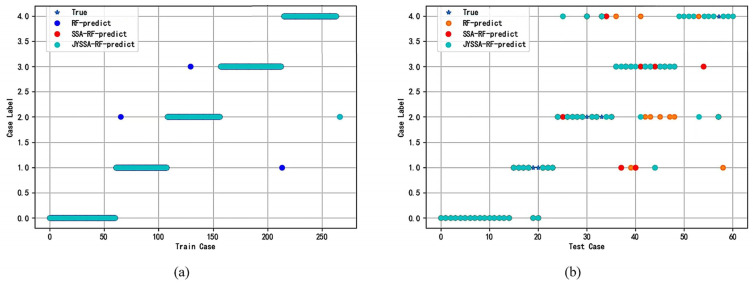

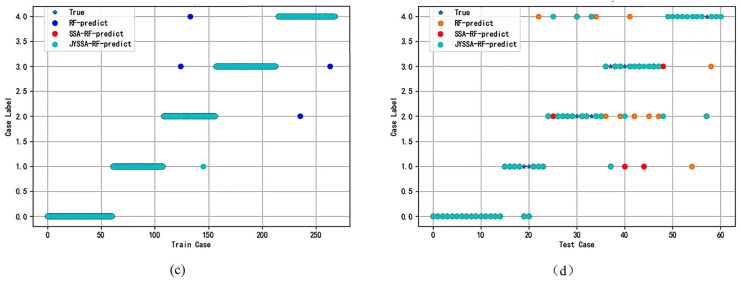
Comparison of the training set and test set: (**a**) training set of 462 bands; (**b**) test set of 462 bands; (**c**) training set of 186 bands; (**d**) test set of 186 bands.

The ordinary RF model was constructed using an RF classifier with n_estimators = 5 and max_features = 3 in the training classification mode. The SSA and JYSSA algorithms’ optimized parameters are reflected in Section 2.3. The comparison of the three algorithms of the test set and training set under 462 bands and 186 bands is shown in Figure 11, and the training set prediction results are represented in different legends to judge whether the training set is accurate with respect to the coverage degree of scattering and the true value, where the blue pentagram represents the true value label, and the blue dots, red dots, and green dots represent the training set prediction of the RF, SSA-RF, and JYSSA-RF models, respectively. The yellow, red, and green dots represent the test sets of the RF, SSA-RF, and JYSSA-RF models, respectively.

To see the round-point coverage of the training set and test set more clearly, the full 462 bands were used as inputs; Table 4 shows the test set validation results and the accuracy of the three models for the full 462 bands. Table 5 shows the test set validation results and the accuracy of the three models with 186 bands as the inputs. For better identification of the procedure, A1, A2, A3, A4, and A5 are defined as 0, 1, 2, 3, and 4, respectively.

### 3.5. Application Validation 

Fourteen grains were reselected from the healthy, mild, moderate, heavier, and heavy mildew groups. To verify the accuracy of the models, the comparison operation described in Section 2.4 was repeated. The results of the three models for 462 bands are shown in Table 6. The results of the three models for 186 bands are shown in Table 7.

**Figure 12:**
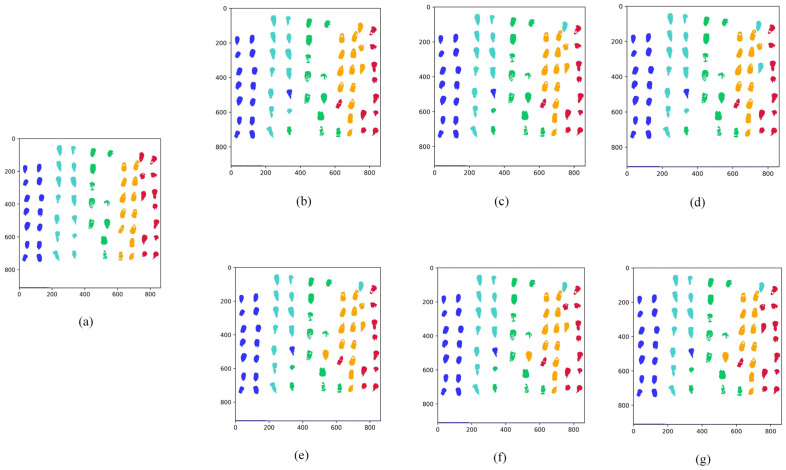
Visualization of predicted moldy maize seeds. (**a**) Different mold visualization images; (**b**) 462-band RF model prediction visualization map; (**c**) 462-band SSA-RF model prediction visualization map; (**d**) 462-band JYSSA-RF model prediction visualization map; (**e**) 186-band RF model prediction visualization map; (**f**) 186-band SSA-RF model prediction visualization map; (**g**) 186-band JYSSA-RF model prediction visualization map.

For better visualization of the predictive ability of the model, Figure 12 shows the visualization of the seed prediction and distinguishes it by different colors, where columns one and two represent A1 in blue, columns three and four represent A2 in cyan, columns five and six represent A3 in green, columns seven and eight represent A4 in yellow, and columns nine and ten represent A5 in red. (a) shows the true label value of the original image, while panels (b), (c), and (d) show the RF model, SSA-RF model, and the JYSSA-RF model, respectively, for the seed prediction under the full waveform. From the prediction images, the prediction effect for heavy mildew is not very good. Panels (e), (f), and (g) show the predictions of the three models under 186 bands, respectively, and it can be seen that the prediction effect for heavy mildew is significantly improved.

## 4. Discussion

Using the proposed algorithm, seeds with different degrees of mildew can be nondestructively tested for hyperspectral mildew. The reflectance value from seed hyperspectral imaging was obtained using a lightweight machine learning model suitable for subsequent transplantation to smartphones or other sensor devices. A faster SSA algorithm can be achieved using this method. The device is highly portable, highly efficient, and has a high level of precision.

While the algorithm achieved the required accuracy, it still needs to be improved. First and foremost, in terms of the SSA itself, although the improved SSA shows reasonable accuracy on the whole, there are ways in which the algorithm itself could be enhanced in terms of its accuracy and convergence speed, such as by using mathematical formulae, applying distributions, and introducing the concepts of sine and cosine, in order to further enhance the accuracy and convergence speed of the SSA. Additionally, the overall algorithm development framework is based on a single thread, from which subsequent multi-threaded development can be carried out to increase the processing efficiency of hyperspectral images. Furthermore, although the images of the hyperspectral imaging system are preprocessed, its advantageous 3D superpixel information has not been used to further explore the algorithm in terms of its application in computer vision processing.

## 5. Conclusions

A hyperspectral imaging method was used to identify the types of mold growing on mold-covered maize seeds. As observed, the spectra of maize seeds with various molds showed substantial variation from one another, and the 500–700 nm reflectance of the spectrum became increasingly degraded with time. At 700–900 nm, an accurate representation of the level of maize seed mold could be found in the steady decline in the absorption process of the peak. There were two major results of this study: an improved SSA and an improved moldy seed prediction and classification algorithm.

Through the implementation of the elite reverse strategy, the SSA was enhanced, and the RF classification model was refined, improving the convergence speed of the optimized JYSSA method and increasing the accuracy of optimal solution judgment from 0.94 to 0.96.

The JYSSA-RF classification model is constructed after the feature band selection, and its accuracy is higher than that of the JYSSA-RF model under the full band, as well as that of the RF and SSA models, with strong prospects for practical applications.

To classify corn seeds with different degrees of mold, this study optimized an RF classifier based on hyperspectral imaging technology and an optimization algorithm. Furthermore, for the first time, the SSA was applied for nondestructive testing, with the potential to improve the characteristic bands and integrate them, along with their associated models, into the equipment in the future. This study presents additional prospects in the field of food security, where its applications could be more varied.

The SSA still needs further research, despite the idea of dividing elites into groups. Some improvements could be made in image preprocessing, image analysis, and identifying seeds with no mildew.

Our future research will not only improve the selection of classification models but also employ 3D superpixels as input sources. This will enable us to further explore the images and optimize the SSA. The wavelength characteristics of mildewed seeds will be further studied in the future.

## Figures and Tables

**Table 1:** Data on different degrees of mildew in maize seeds.

Seed Number	Degree of Mold and Mildew	Number of Seeds
A1	Healthy	77
A2	Mild mildew	56
A3	Moderate mold	63
A4	Heavier mold	70
A5	Heavy mold	70

**Table 2:** Optimal parameters of 462-band SSA and JYSSA.

Algorithm	Optimal Adaptation	N_Estimators Optimal Solution	Max_Features Optimal Solution
SSA	0.151	14	139
JYSSA	0.151	41	100

**Table 3:** Optimal parameters of 186-band SSA and JYSSA.

Algorithm	Optimal Adaptation	N_Estimators Optimal Solution	Max_Features Optimal Solution
SSA	0.155	31	47
JYSSA	0.147	25	96

**Table 4:** Results of the three model test sets in the 462 bands.

Models	Seed Tags	Precision	Recall	Sample Size	Accuracy
JYSSA-RF	0	0.88	1.00	15	0.85
	1	0.78	0.78	9	
	2	0.67	0.50	12	
	3	0.79	0.85	13	
	4	0.67	0.67	12	
SSA-RF	0	0.88	1.00	15	0.85
	1	0.78	0.78	9	
	2	0.90	0.75	12	
	3	1.00	0.77	13	
	4	0.73	0.92	12	
RF	0	0.88	1.00	15	0.77
	1	0.78	0.78	9	
	2	0.89	0.67	12	
	3	1.00	0.85	13	
	4	0.73	0.92	12	

**Table 5:** Results of the three model test sets in the 186 bands.

Models	Seed Tags	Precision	Recall	Sample Size	Accuracy
JYSSA-RF	0	0.88	1.00	15	0.85
	1	0.60	0.67	9	
	2	0.70	0.58	12	
	3	0.88	0.54	13	
	4	0.62	0.83	12	
SSA-RF	0	0.88	1.00	15	0.85
	1	0.78	0.78	9	
	2	0.82	0.75	12	
	3	1.00	0.77	13	
	4	0.79	0.92	12	
RF	0	0.93	1.00	14	0.74
	1	0.93	0.93	14	
	2	1.00	0.93	14	
	3	1.00	0.93	14	
	4	0.93	1.00	14	

**Table 6:** Validation set results of the three varieties of models with 462 bands.

Models	Seed Tags	Precision	Recall	Sample Size	Accuracy
JYSSA-RF	0	0.93	1.00	14	0.94
	1	0.93	0.93	14	
	2	1.00	0.86	14	
	3	0.92	0.86	14	
	4	0.88	1.00	14	
SSA-RF	0	0.93	1.00	14	
	1	0.93	1.00	14	0.93
	2	0.81	0.93	14	
	3	1.00	0.86	14	
	4	1.00	0.77	14	
RF	0	0.93	1.00	14	
	1	0.93	0.93	14	
	2	1.00	0.93	14	0.91
	3	1.00	0.93	14	
	4	0.93	1.00	14	

**Table 7:** Validation set results of the three varieties of models with 186 bands.

Models	Seed Tags	Precision	Recall	Sample Size	Accuracy
JYSSA-RF	0	0.93	1.00	14	0.96
	1	0.93	0.93	14	
	2	0.92	0.86	14	
	3	1.00	0.86	14	
	4	0.81	0.93	14	
SSA-RF	0	0.93	1.00	14	
	1	0.93	0.93	14	0.94
	2	1.00	0.86	14	
	3	0.93	0.93	14	
	4	0.93	1.00	14	
RF	0	0.93	1.00	14	
	1	0.93	0.93	14	
	2	1.00	0.93	14	0.91
	3	1.00	0.92	14	
	4	0.93	1.00	14	

## Data Availability

Not applicable.
